# Anesthetic Management of Delayed Primary Arterial Switch Operation in a Two-Month-Old Infant With Dextro-Transposition of the Great Arteries (d-TGA), Preserved Left Ventricular Mass, and Patent Ductus Arteriosus: A Case Report

**DOI:** 10.7759/cureus.115537

**Published:** 2026-08-31

**Authors:** Ayham Khddam, Laila Tahseen Turkmani, Mohammad Y Hajeer

**Affiliations:** 1 Department of Anesthesia and Resuscitation, Children's Hospital, University of Damascus, Damascus, SYR; 2 Department of Orthodontics, Faculty of Dentistry, University of Damascus, Damascus, SYR

**Keywords:** delayed arterial switch (aso), milrinone, patent ductus arteriosus (pda), pediatric anesthesia, transposition of the great arteries (d‑tga)

## Abstract

For dextro-transposition of the great arteries (d-TGA) with an intact ventricular septum, the arterial switch operation (ASO) is usually performed early to avoid left ventricular (LV) deconditioning. When infants present late, the risk of postoperative LV failure rises substantially. In this case report, we describe a two‑month‑old infant (3.98 kg) with d‑TGA, severe hypoxemia (peripheral oxygen saturation (SpO_2_) 49%), and failure to thrive. Despite late presentation, LV mass and its circular geometry were maintained (ejection fraction 85.1%) in association with a large, nonrestrictive 6.1 mm patent ductus arteriosus (PDA). A primary ASO was completed successfully. Anesthesia was managed with a total intravenous anesthesia technique using high-dose opioids. During separation from cardiopulmonary bypass, milrinone (0.5 mcg/kg/min) and low-dose epinephrine (0.05 mcg/kg/min) were administered to enhance myocardial contractility while optimizing the balance between systemic and pulmonary vascular resistances (SVR and PVR) to prevent low cardiac output syndrome and reactive pulmonary hypertensive crises. The patient remained hemodynamically stable postoperatively. In this selected late-presenting infant, primary ASO was feasible alongside preserved LV mass and geometry in the presence of a large PDA. Comprehensive preoperative echocardiography and tailored anesthetic management focusing on biventricular loading and PVR/SVR balance are key to achieving favorable outcomes.

## Introduction

The arterial switch operation (ASO) is still the gold standard for treating dextro-transposition of the great arteries (d-TGA), which accounts for about 5%-7% of congenital heart disease [1,2]. Outcomes are poor, with high early neonatal mortality without timely anatomical correction to restore physiologic in-series biventricular circulation [3].

From a physiological perspective, pulmonary vascular resistance (PVR) drops steeply after birth. As this occurs, the left ventricle (LV) gradually regresses and remodels so that the second to third week of life represents a critical period associated with progressive LV deconditioning and significantly increased risk of acute postoperative LV failure, rather than an absolute temporal cutoff. The primary ASO may be performed after this time but may lead to acute postoperative LV failure, which may be irreversible. Traditionally, this has meant staged repair, most commonly pulmonary artery banding followed by late ASO [2,4].

A large, nonrestrictive patent ductus arteriosus (PDA) permits bidirectional intercirculatory flow, where the magnitude of pressure transmission and potential equalization depends on ductal diameter, shunt volume, and the relative ratio of pulmonary to systemic vascular resistance (PVR/SVR). In d-TGA, an unrestricted duct can effectively “condition” the LV by maintaining it at systemic pressures, which helps in preserving myocardial mass, wall thickness, and geometry instead of the expected postnatal regression. Meanwhile, the duct provides for adequate mixing between the two parallel circulations, which is vital for systemic oxygenation and survival. Herein, we describe the anesthetic and perioperative management of a late-presenting two-month-old infant with d-TGA and an intact ventricular septum who successfully underwent high-risk primary ASO without the need for extracorporeal membrane oxygenation (ECMO).

## Case presentation

Preoperative evaluation and pathophysiologic synthesis

A two-month-old female infant (3.98 kg) presented with persistent central cyanosis since birth and marked failure to thrive. On examination, she was deeply cyanotic with a peripheral oxygen saturation (SpO_2_) of 49% on room air. Serial arterial blood gases showed severe hypoxemia, with a baseline arterial partial pressure of oxygen (PaO_2_) of 28 mmHg. However, clinically and metabolically, she was remarkably stable, with no cardiogenic shock, metabolic acidosis, or neurologic sequelae. Her survival seemed to represent several coordinated compensatory mechanisms. *Active Secondary Polycythemia*

Chronic hypoxemia probably raised renal erythropoietin, leading to secondary polycythemia (hemoglobin 16.1 g/dL, hematocrit 49.7%) [5], improving oxygen-carrying capacity. Platelet count was stable (154,000/mm^3^), and coagulation was preserved, with an international normalized ratio (INR) of 1.0. *Fetal Hemoglobin (HbF) Retention*

Persistence of fetal hemoglobin (HbF) at two months of age-a recognized compensatory physiological adaptation to chronic cyanotic hypoxia-would favor oxygen loading in the lungs and intercirculatory mixing zones, thereby supporting tissue oxygenation and organ survival under severe hypoxic stress [6]. *Hemodynamic Mixing by a Large PDA*

The 6.1 mm nonrestrictive PDA functioned as a high-caliber conduit enabling continuous, high-volume bidirectional intercirculatory mixing between the parallel circulations. Continuous-wave Doppler interrogation confirmed bidirectional ductal shunting with a peak velocity of 2.0 m/s, supporting a low gradient across the ductus between the parallel circulations, allowing organs to receive partially oxygenated blood. There were no signs of severe anaerobic metabolism: renal function and electrolytes were stable, pH was compensated (7.32), PaCO_2_ was 32 mmHg (consistent with compensatory hyperventilation), and serum lactate was mildly elevated at 2.8 mmol/L. Normal leukocyte counts and inflammatory markers argued against systemic infection. Mild elevation of aspartate aminotransferase (AST) (70 U/L) was suggestive of some hepatic congestion due to chronic strain. A brief and benign episode of calcium oxalate crystalluria was also noted.

Evidence of a compensatory hyperdynamic state with tachycardia (HR 160 bpm) and a respiratory rate of 32/min supported cardiac output. On examination, there was a prominent continuous murmur consistent with PDA flow. Cerebral vasodilation in chronic hypoxia probably shunted more blood to the brain, offering some protection against hypoxic-ischemic injury [7,8]. The overall patient was assigned to American Society of Anesthesiologists (ASA) physical status IV [9]. The clinical picture led the team to go directly to primary ASO without balloon atrial septostomy (BAS).

Assessment of echocardiography

Transthoracic echocardiography confirmed d-TGA with ventriculoarterial discordance, atrioventricular concordance, and right-sided chamber enlargement consistent with chronic volume overload (Table 1). Preoperative transthoracic echocardiography demonstrated a non-restrictive patent foramen ovale (PFO/atrial septal communication measuring 4.2 mm) with bidirectional atrial shunting. The combination of this adequate interatrial communication and the large 6.1 mm PDA facilitated effective intercirculatory mixing. In the absence of severe hemodynamic instability, profound tissue acidosis, or restrictive atrial septum, emergency balloon atrial septostomy (BAS) was not required, allowing the surgical team to proceed directly to definitive primary ASO.

**Table 1:** Preoperative baseline clinical, laboratory, and echocardiographic parameters. ABG, arterial blood gas; AST, aspartate aminotransferase; Cl⁻, chloride; EF, ejection fraction; FS, fractional shortening; Hb, hemoglobin; Hct, hematocrit; HR, heart rate; INR, international normalized ratio; IVSd, diastolic interventricular septal thickness; K⁺, potassium; LV, left ventricle/left ventricular; LVEDD, left ventricular end-diastolic diameter; LVESD, left ventricular end-systolic diameter; LVMI, left ventricular mass index; LVPWd, diastolic left ventricular posterior wall thickness; Na⁺, sodium; PaCO₂, arterial partial pressure of carbon dioxide; PaO_2_, arterial partial pressure of oxygen; PDA, patent ductus arteriosus; RR, respiratory rate; SpO_2_, peripheral oxygen saturation; TR, tricuspid regurgitation; WBC, white blood cell.

Assessment Category	Parameter	Preoperative Value	Pediatric Reference Value (Age 2 Months)
Vital Signs & Physical Exam	Peripheral Oxygen Saturation (SpO_2_)	49%	95% – 100%
	Heart Rate (HR)	160 beats/min	100 – 160 beats/min
	Respiratory Rate (RR)	32 breaths/min	30 – 50 breaths/min
	Cardiac Auscultation	Prominent continuous murmur	Normal S1/S2; no pathological murmur
Hematology & Coagulation	Hemoglobin (Hb)	16.1 g/dL	10.5 – 13.5 g/dL
	Hematocrit (Hct)	49.7%	31% – 41%
	Platelet Count	154,000/mm^3^	150,000 – 450,000 /mm^3^
	International Normalized Ratio (INR)	1.0	0.8 – 1.2
Organ Function & Urine	Serum Creatinine	0.3 mg/dL	0.2 – 0.4 mg/dL
	Serum Urea	12 mg/dL	5 – 18 mg/dL
	Serum Sodium (Na⁺)	138 mmol/L	135 – 145 mmol/L
	Serum Potassium (K⁺)	4.2 mmol/L	3.5 – 5.3 mmol/L
	Serum Chloride (Cl⁻)	101 mmol/L	98 – 107 mmol/L
	Aspartate Aminotransferase (AST)	70 U/L	15 – 55 U/L
	White Blood Cell (WBC) Count	9,200/mm^3^	6,000 – 17,500/mm^3^
	Routine Urinalysis	Calcium oxalate crystalluria	Negative for crystals/protein/blood
Arterial Blood Gas (ABG)	Arterial pH	7.32	7.35 – 7.45
	Arterial CO_2_ Partial Pressure (PaCO_2_)	32 mmHg	35 – 45 mmHg
	Arterial O_2_ Partial Pressure (PaO_2_)	28 mmHg	80 – 100 mmHg
	Serum Lactate	2.8 mmol/L	0.5 – 2.0 mmol/L
Echocardiographic Parameters	Patent Ductus Arteriosus (PDA) Diameter	6.1 mm	0 mm (Closed postnatally)
	Tricuspid Regurgitation (TR) Jet Velocity	3.84 m/s	< 2.5 m/s
	LV End-Diastolic Diameter (LVEDD)	2.25 cm	1.8 – 2.3 cm
	LV End-Systolic Diameter (LVESD)	1.08 cm	1.0 – 1.4 cm
	Ejection Fraction (EF)	85.1%	55% – 75%
	Fractional Shortening (FS)	51.9%	28% – 42%
	Diastolic LV Posterior Wall Thickness (LVPWd)	4.5 mm	3.0 – 4.5 mm
	Diastolic Interventricular Septal Thickness (IVSd)	4.4 mm	3.0 – 4.5 mm
	Left Ventricular Mass Index (LVMI)	57.9 g/m^2^	35 – 50 g/m^2^

**Figure 1:**
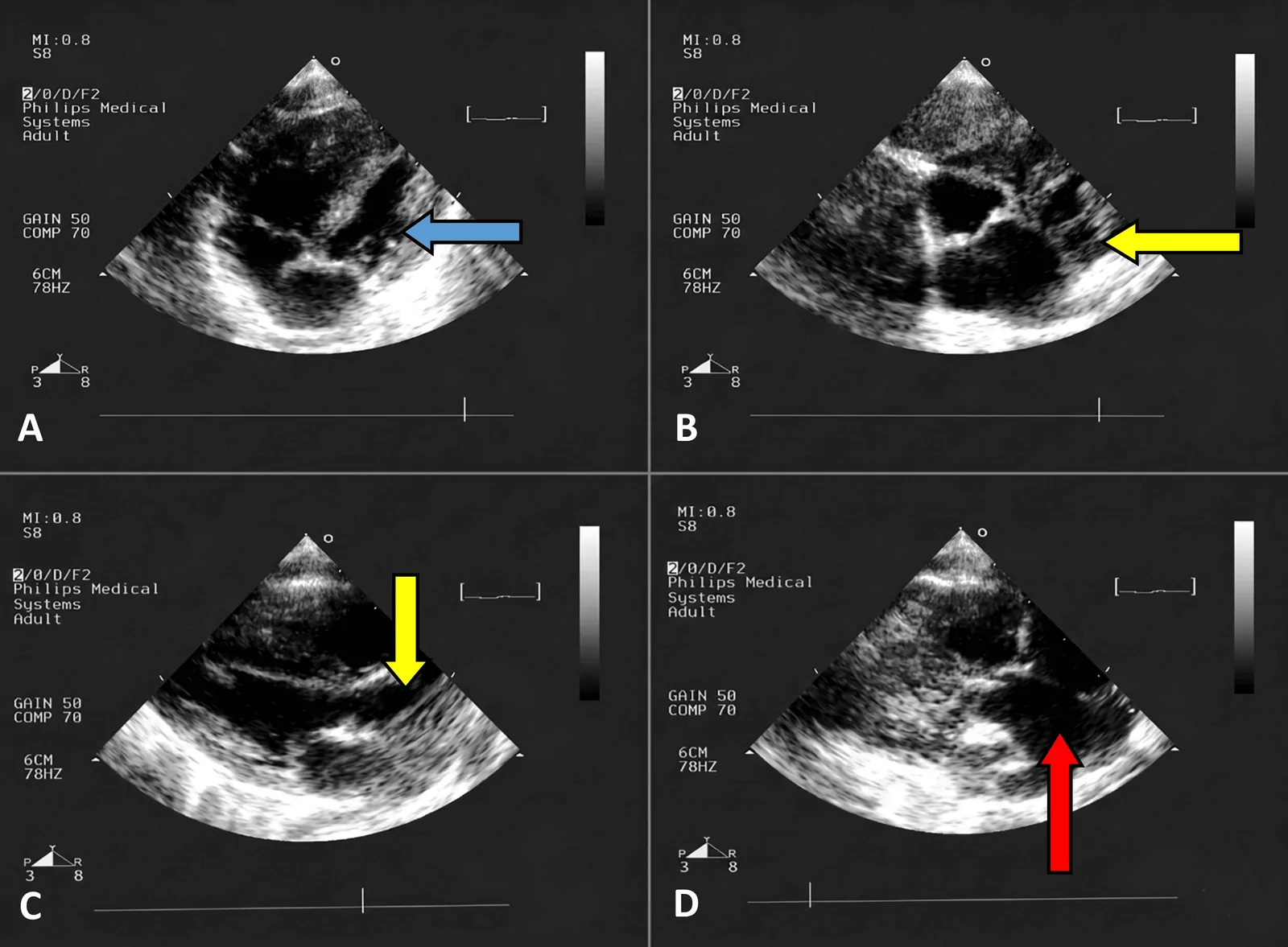
Preoperative transthoracic echocardiography of d-TGA anatomy. A: Apical four-chamber view demonstrating atrioventricular concordance alongside marked dilatation of the right-sided chambers as indicated by the blue arrow. B and C: Views depicting the classic parallel (non-crossing) outflow tracts, pathognomonic for dextro-transposition of the great arteries (d-TGA) as indicated by the yellow arrows. D: Parasternal short-axis view demonstrating a well-conditioned, circular ("O-shaped") left ventricle with no interventricular septal flattening indicated by the red arrow, confirming preserved ventricular geometry suitable for immediate systemic afterload.

Preoperative echocardiography demonstrated systemic-level right ventricular (RV) systolic pressure, evidenced by a tricuspid regurgitation (TR) jet velocity of 3.84 m/s (peak gradient 59.0 mmHg), which corresponds to systemic arterial pressure in d-TGA where the RV supports systemic circulation. Near-equalization of pulmonary arterial pressure with systemic pressure was inferred from the low peak systolic gradient across the nonrestrictive 6.1 mm PDA (peak velocity 2.0 m/s; estimated peak gradient ~16 mmHg), alongside preserved circular LV geometry ("O-shape"; Figure 1).

**Figure 2:**
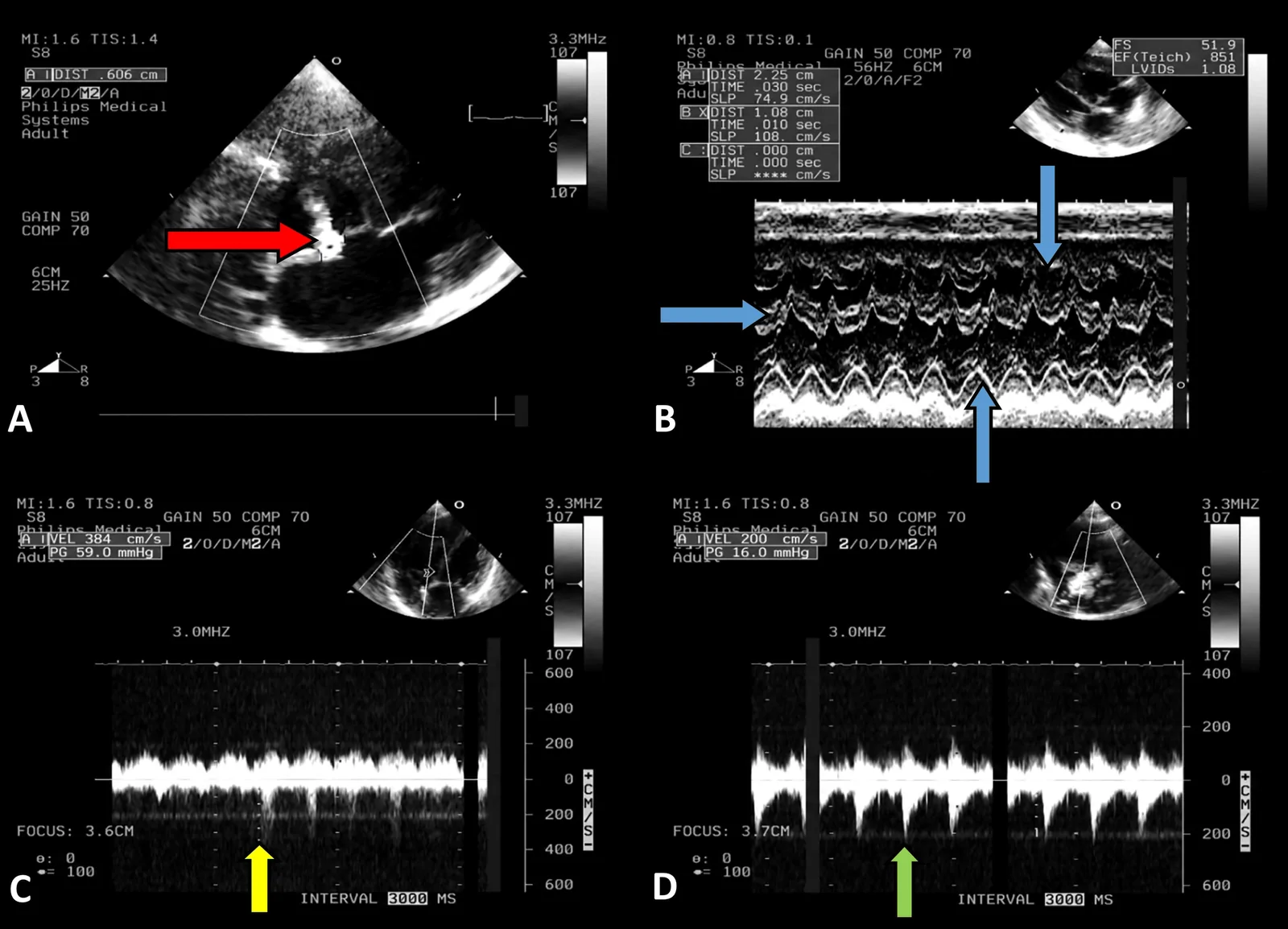
Preoperative echocardiographic assessment of left ventricular anatomy and hemodynamics. A: Two-dimensional parasternal short-axis view showing a large, non-restrictive PDA measuring 6.1 mm in diameter, indicated by the red arrow, permitting critical intercirculatory mixing. B: M-mode evaluation of the left ventricle demonstrating hyperdynamic baseline systolic function indicated by blue arrows marking measurement calipers. C: Continuous-wave Doppler interrogation of the tricuspid regurgitation jet revealing a peak velocity of 3.84 m/s (indicated by the yellow arrow), consistent with severe preoperative pulmonary hypertension. D: Continuous-wave Doppler of ductal flow showing a peak velocity of 2.0 m/s (indicated by the green arrow), confirming continuous pressure equalization.

M-mode imaging (Figure 2) showed hyperdynamic LV systolic function with ejection fraction (EF) 85.1%, fractional shortening (FS) 51.9%, left ventricular end-diastolic diameter (LVEDD) 2.25 cm, and left ventricular end-systolic diameter (LVESD) 1.08 cm. Left ventricular mass index (LVMI) was 57.9 g/m^2^, and left ventricular diastolic posterior wall thickness (LVPWd) was 4.5 mm. These values were above commonly used thresholds for postoperative LV adequacy (LVPWd ≥4.0 mm; LVMI ≥35-50 g/m^2^). The team selected primary ASO without staged palliation.

Stages of anesthesia

Anesthetic induction aimed to prevent sympathetic surges or crying that could trigger a sudden spike in pulmonary vascular resistance (PVR) and exacerbate right-to-left shunting [1]. We slowly titrated intravenous fentanyl (10 mcg/kg) and midazolam (0.2 mg/kg), supplemented with low-concentration sevoflurane in 100% oxygen. Atracurium (0.5 mg/kg) provided neuromuscular blockade for endotracheal intubation. For continuous monitoring and drug delivery, we placed a right femoral arterial line, a right internal jugular double-lumen central venous catheter, and a left femoral venous line.

Anesthesia maintenance relied on total intravenous anesthesia (TIVA) with continuous infusions of fentanyl (2-5 mcg/kg/hr) and midazolam (0.1 mg/kg/hr) to avoid the direct myocardial depressant effects of volatile agents. Perioperative management focused on balancing systemic vascular resistance (SVR) and PVR. Mechanical ventilation was adjusted to maintain mild hyperventilation (PaCO_2_, 30 - 35 mmHg) with FiO_2_ titrated up to 1.0 as clinically required. This strategy controlled PVR without inducing severe hypocapnia or hyperoxia, which could provoke cerebral vasoconstriction or systemic-to-pulmonary steal. Serial arterial blood gas analyses guided ventilatory and acid-base adjustments.

Before bypass cannulation, systemic anticoagulation was established with heparin (300 IU/kg) to target an activated clotting time (ACT) above 480 seconds. Peak ACT reached 846 seconds prior to CPB initiation and remained above 503 seconds throughout the 185-minute bypass run. Moderate-to-deep hypothermia at 22°C was maintained during an aortic cross-clamp duration of 117 minutes. Intermittent cold blood cardioplegia delivered into the aortic root every 20-30 minutes provided myocardial protection [10-12].

Weaning from CPB required careful adjustment of right and left ventricular afterload. During rewarming, we initiated an inodilator infusion of milrinone (0.5 mcg/kg/min) combined with low-dose epinephrine (0.05 mcg/kg/min). This regimen supported myocardial contractility while lowering PVR, preventing post-bypass pulmonary hypertensive crises or low cardiac output syndrome. Post-bypass modified ultrafiltration (MUF) was performed to reduce tissue edema and concentrate hematocrit [13]. Heparin was reversed with slow intravenous protamine (1.0-1.3 mg per 100 IU heparin) under direct pressure monitoring, followed by tranexamic acid (10 mg/kg) to minimize postoperative bleeding.

Postoperative pediatric intensive care unit course, discharge, and follow-up

We moved her to the PICU on a ventilator right after surgery. For the first 24 hours, we kept her deeply sedated on synchronized intermittent mandatory ventilation (SIMV) with good pain control. We did this to give her brain and metabolism some time to recover while keeping the workload on her heart low and protecting her LV compliance. Fortunately, she didn't need ECMO or high-frequency oscillatory ventilation (HFOV) at all. We tapered off her vasoactive drugs fairly quickly, and we were able to extubate her to room air at 48 hours.

We discharged her home in stable condition on postoperative day 10. By then, she was active, feeding well by mouth, and only needed a low-dose prophylactic oral diuretic.

Looking at the labs, her tissue acidosis was completely gone, and her lactate came down to 1.5 mmol/L. Her secondary polycythemia also cleared up, with her hemoglobin and hematocrit settling at 13.8 g/dL and 40%. Her cyanosis was completely gone, and her oxygen saturations on room air stabilized between 93% and 100% (Table 2).

**Table 2:** Longitudinal comparison of clinical, laboratory, and echocardiographic parameters. ABG, arterial blood gas; EF, ejection fraction; FS, fractional shortening; Hb, hemoglobin; Hct, hematocrit; HR, heart rate; IVSd, diastolic interventricular septal thickness; LV, left ventricle; LVEDD, left ventricular end-diastolic diameter; LVPWd, diastolic left ventricular posterior wall thickness; PaCO_2_, arterial partial pressure of carbon dioxide; PaO_2_, arterial partial pressure of oxygen; PDA, patent ductus arteriosus; POD, postoperative day; RR, respiratory rate; RV, right ventricle; SpO_2_, peripheral oxygen saturation; TR, tricuspid regurgitation.

Category	Parameter	Preoperative Value	Postoperative Value (POD 10 - Discharge)	Outpatient Follow-up Value (Week 4)	Pediatric Reference Value
Vital Signs & ABG	Peripheral Oxygen Saturation (SpO_2_)	49%	98% (Room air)	99% (Room air)	95% – 100%
	Heart Rate (HR)	160 beats/min	122 beats/min	115 beats/min	100 – 160 beats/min
	Respiratory Rate (RR)	32 breaths/min	36 breaths/min	32 breaths/min	30 – 50 breaths/min
	Arterial pH	7.32	7.40	7.42	7.35 – 7.45
	Arterial PaO_2_	28 mmHg	88 mmHg	94 mmHg	80 – 100 mmHg
	Arterial PaCO_2_	32 mmHg	38 mmHg	40 mmHg	35 – 45 mmHg
Laboratory Findings	Hemoglobin (Hb) level	16.1 g/dL	13.8 g/dL	12.5 g/dL	10.5 – 13.5 g/dL
	Hematocrit (Hct)	49.7%	40.0%	37.0%	31% – 41%
	Serum Lactate	2.8 mmol/L	1.5 mmol/L	1.1 mmol/L	0.5 – 2.0 mmol/L
Echocardiography	PDA Diameter	6.1 mm	0 mm (Ligated)	0 mm (No residual flow)	0 mm
	TR Jet Velocity	3.84 m/s	2.1 m/s	1.9 m/s	< 2.5 m/s
	RV Peak Pressure Gradient	59 mmHg	23 mmHg	19 mmHg	< 25 mmHg
	LV End-Diastolic Diameter (LVEDD)	2.25 cm	2.10 cm	1.88 cm	1.8 – 2.3 cm
	Ejection Fraction (EF)	85.1%	65.0%	88.7%	55% – 75%
	Fractional Shortening (FS)	51.9%	36.0%	56.0%	28% – 42%
	Diastolic Posterior Wall Thickness (LVPWd)	4.5 mm	4.5 mm	5.8 mm	3.0 – 4.5 mm
	Diastolic Interventricular Septal Thickness (IVSd)	4.4 mm	4.6 mm	4. 9 mm	3.0 – 4.5 mm
	Neo-pulmonary Artery Outflow	No baseline obstruction	Peak velocity < 1.8 m/s (Gradient 19 mmHg)	Peak velocity < 1.6 m/s (No branch stenosis)	Peak velocity < 1.8 m/s

Her follow-up TTE (done at discharge and at week 4) showed excellent results. First, the PDA was completely ligated (0 mm) with zero residual shunt. Second, her pulmonary pressures were much lower, with the RV systolic pressure dropping significantly-her tricuspid regurgitation (TR) jet velocity was 2.1 m/s, with a peak gradient of 23 mmHg (a massive drop from the preoperative 59 mmHg). Her LV was adapting well to the systemic workload: LVEDD was 2.1 cm, posterior wall thickness was 4.5 mm, and she had normal systolic function (EF 65%, FS 36%). Crucially, we saw no regional wall motion abnormalities, which confirmed her coronaries were patent after the transfer (we used medially based rectangular flaps/the Melbourne trapdoor technique). Finally, velocities across the reconstructed pulmonary branches were completely normal (peak <1.8 m/s; gradient 19 mmHg), confirming a successful LeComte maneuver (Table 3).

**Table 3:** Detailed chronological timeline of the patient's clinical and laboratory course.

Timeline/Stage	Clinical Events & Management	Key Hemodynamic & Clinical Outcomes
Admission Day (Day -1)	• Admission of the two-month-old infant (weight: 3.98 kg). • Performed baseline laboratory tests and transthoracic echocardiogram (TTE).	• Severe central cyanosis (SpO_2_ of 49%). • Confirmed diagnosis of d-TGA with a large PDA (6.1 mm) and conditioned left ventricle (LVMI of 57.9 g/m^2^).
Surgical Day (Day 0)	• Performed primary arterial switch operation (ASO), LeCompte maneuver, and coronary translocation. • Successfully weaned from cardiopulmonary bypass (CPB) and transferred to the pediatric ICU (PICU).	• CPB time of 185 minutes; aortic cross-clamp time of 117 minutes. • Achieved hemodynamic stability post-modified ultrafiltration (MUF) and heparin reversal with protamine without requiring ECMO support.
ICU Course (Days 1–2)	• Maintained mechanical ventilation and multimodal analgesia. • Gradual weaning of vasoactive/inotropic support and cessation of deep sedation. • Successful tracheal extubation on postoperative day 2.	• Maintained excellent ventricular compliance and successfully avoided reactive pulmonary hypertensive crises. • Preserved biventricular systolic performance with stable SpO_2_ (93% to 100%) on room air.
Ward Course (Days 3–9)	• Transferred patient to the pediatric cardiac surgery ward. • Initiated gradual oral feeding and prophylactic oral diuretics.	• Excellent tolerance to oral intake with progressive weight gain. • Normal healing of the median sternotomy incision, with no signs of heart failure or pericardial effusion.
Discharge Day (Day 10)	• Performed final clinical examination and predischarge echocardiogram. • Discharged home on a low-dose prophylactic oral diuretic regimen.	• Stable clinical status with complete resolution of tissue acidosis (serum lactate of 1.5 mmol/L). • Echocardiography confirmed complete ductal closure (0 mm), normal systolic function (EF of 65%), and no branch pulmonary stenosis.
Outpatient Follow-up (Week 4)	• Outpatient clinic consultation and routine follow-up transthoracic echocardiogram.	• Excellent clinical status and age-appropriate physical growth (weight: 4.35 kg). • Echocardiography verified patent and well-perfused translocated coronary arteries with laminar flow across the great vessels.

**Figure 3:**
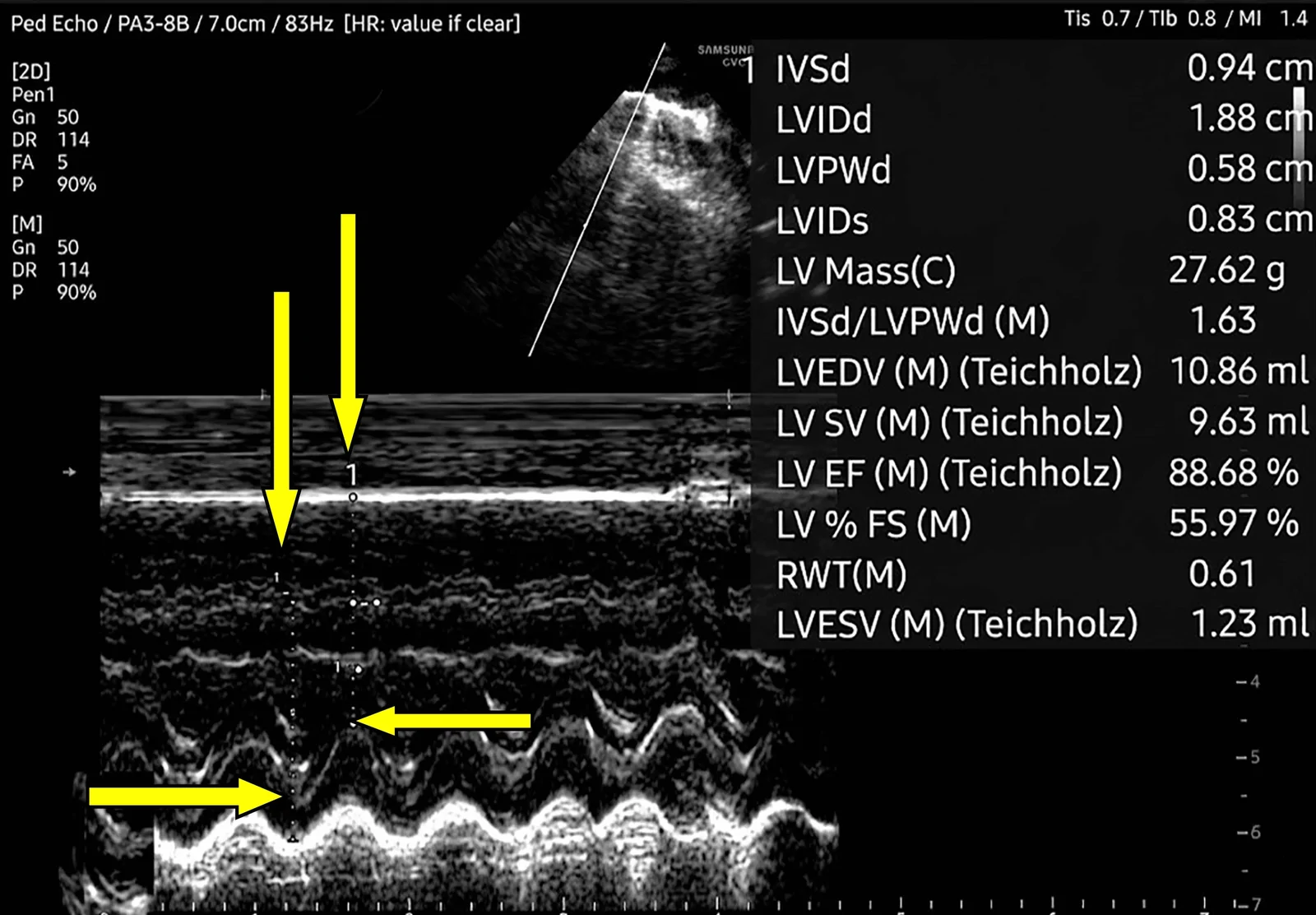
Postoperative M-mode echocardiographic evaluation of the left ventricle at three to four weeks follow-up. Parasternal short-axis M-mode view obtained three to four weeks postoperatively, demonstrating successful left ventricular adaptation to systemic workload. Measurement calipers (indicated by the yellow arrows) highlight a hyperdynamic left ventricle with an ejection fraction of 88.7% and fractional shortening of 56.0%. Diastolic interventricular septal thickness (IVSd: 0.49 cm/4.9 mm; indicated by the top caliper arrow) and diastolic left ventricular posterior wall thickness (LVPWd: 0.58 cm/5.8 mm; indicated by the bottom caliper arrow) confirm robust myocardial mass without regressive atrophy. EF, ejection fraction; FS, fractional shortening; SV, stroke volume; RWT, relative wall thickness; IVSd, diastolic interventricular septal thickness; LVIDd, diastolic left ventricular internal diameter; LVPWd, diastolic left ventricular posterior wall thickness.

When we saw her at the four-week outpatient clinic, she was doing great. She was growing well, with her weight up to 4.35 kg. Most importantly, her translocated coronaries were patent and well-perfused with nice, laminar flow (Figure 3).

## Discussion

Late-presenting d-TGA and the role of PDA in LV conditioning

Primary arterial switch operation (ASO) for d-transposition of the great arteries (d-TGA) is extremely time-dependent and is usually limited to the first few weeks of life. When an infant presents at two months of age with uncorrected d-TGA, treatment becomes particularly challenging. The literature consistently advises against performing a primary ASO after the fourth week because of the substantial risk of sudden, severe postoperative left ventricular (LV) failure [14]. In our patient, however, a continuous, non-restrictive patent ductus arteriosus (PDA) effectively acted like an intrinsic pulmonary artery band (PAB). That physiologic “conditioning” was central to our decision to proceed with a single-stage definitive anatomical repair rather than a staged palliative approach.

In late presenters, the presence of a large PDA is a key factor in maintaining LV mass and function [14]. Normally after birth, pulmonary vascular resistance (PVR) falls quickly, and the LV progressively thins and regresses. A non-restrictive PDA interrupts this regressive remodeling and helps preserve the myocardial mass needed to support systemic circulation after ASO [15]. This can be understood as the result of two loading conditions happening at the same time: (i) Pressure overload (afterload): The PDA carries systemic arterial pressure back into the pulmonary artery, so the LV is effectively exposed to a systemic-level afterload, encouraging hypertrophy [16,17]; (ii) Volume overload (preload): The left-to-right shunt boosts pulmonary venous return and increases left atrial volume, activating the Frank-Starling mechanism via higher preload and helping preserve diastolic chamber size [15,16].

Beyond myocardial mass, a non-restrictive PDA also helps preserve normal cardiac geometry. In late-presenting d-TGA with an intact ventricular septum (IVS), an unconditioned LV often loses its usual ellipsoid shape and becomes flattened or “banana-shaped” (D-shaped) as the interventricular septum shifts leftward under systemic right ventricular (RV) pressure. In our case, sustained systemic-level pulmonary pressure allowed the LV to maintain a circular “O” configuration. Echocardiographic confirmation of this circular geometry is considered a reliable clinical sign that the LV is ready to take on systemic work [15,18].

Quantitative echocardiographic safeguards 

Because these cases are high-risk, management needs to be anchored in objective, validated measurements. Determining whether the LV can tolerate a late primary ASO depends on specific echocardiographic indices. Traditionally, an LV mass index (LVMI) above 50 g/m^2^ - based on the criteria described by Lacour-Gayet et al. [19] - has been used as a key threshold. In our assessment, we combined that framework with additional established parameters: a diastolic LV posterior wall thickness (LVPWd) ≥ 4.0 mm and a left-to-right ventricular systolic pressure ratio of at least 0.70 [20].

Ultimately, our operative plan followed these criteria to reduce the risk of postoperative LV failure associated with delayed repair [15,16,18]. We also relied on the infant’s preserved myocardial plasticity and compliance, assuming that chronic mechanical stress had already driven the cellular hypertrophy needed for systemic support after ASO [14]. Follow-up transthoracic echocardiography (TTE) three weeks postoperatively supported this both structurally and functionally (Figure 3). M-mode findings showed strong adaptation to systemic vascular resistance, without myocardial failure, chamber dilation, or reverse remodeling. Postoperative LVPWd was 5.8 mm, and diastolic interventricular septal thickness (IVSd) was 4.9 mm-both clearly above safety cutoffs. The systemic LV also demonstrated vigorous, hyperdynamic function (EF 88.7%, FS 56.0%), confirming successful integration of the conditioned ventricle into systemic circulation.

Surgical and coronary translocation challenges 

In older infants, coronary translocation and reimplantation are technically more demanding. Myocardial hypertrophy and changes in great-vessel geometry from chronic pressure and volume loading increase the risk of coronary torsion or tension. To address these issues, we harvested large coronary buttons and used medially based rectangular flaps (the Melbourne trapdoor technique) to reduce the rotation arc and minimize tension at the coronary anastomoses.

In addition, during delayed ASO, the LeComte maneuver can increase traction on the newly transferred coronary buttons. Since the great vessels at two months are larger and less compliant than in neonates, anterior translocation of the pulmonary bifurcation creates greater spatial displacement of the neo-aorta. This makes careful coronary mobilization and the use of medially based rectangular flaps especially important to avoid kinking or stretching. Even with an uncomplicated immediate result, long-term surveillance with cardiovascular magnetic resonance (CMR) or computed tomography (CT) angiography remains important to detect late branch pulmonary artery stenosis or coronary ostial narrowing [2,3].

Advanced anesthetic management and post-CPB weaning dynamics 

The period immediately after separation from cardiopulmonary bypass (CPB) is the most physiologically vulnerable phase in late-presenting d-TGA. The LV, now assigned to systemic circulation, must abruptly shift from a parallel to an in-series circulation and confront systemic vascular resistance (SVR) for the first time. Although a large PDA helped preserve LV mass preoperatively, prolonged ischemic time (117 minutes of cross-clamp) increased the risk of acute low cardiac output syndrome (LCOS) [21,22]. At the same time, the RV remains susceptible to postoperative pulmonary hypertensive crises due to endothelial activation from bypass and ischemia-reperfusion injury.

To manage these fragile biventricular loading conditions, we used a combined vasoactive strategy: early milrinone (0.5 mcg/kg/min) with low-dose epinephrine (0.05 mcg/kg/min) during rewarming. Earlier pediatric cardiac anesthesia often depended on catecholamine-heavy inotropy (e.g., dopamine or higher-dose epinephrine), which could raise PVR and provoke reactive pulmonary hypertensive episodes [23]. Milrinone, a selective phosphodiesterase-3 inhibitor, functions as an “inodilator,” improving contractility while preferentially dilating the pulmonary vasculature and thereby unloading the RV [24]. Because milrinone can lower systemic vascular tone and potentially reduce coronary perfusion pressure-particularly relevant for newly reimplanted coronaries-we added a very low dose of epinephrine. This pairing supported both ventricles without increasing PVR or triggering tachyarrhythmias and offered a more targeted alternative to single-agent catecholamine strategies [25].

We also deliberately avoided aggressive hyperventilation. Severe hypocapnia (arterial carbon dioxide partial pressure (PaCO_2_) < 25 mmHg) was once promoted as a non-pharmacologic method to reduce PVR in congenital cardiac surgery [26]. More recent evidence cautions that extreme hypocapnia can cause cerebral vasoconstriction, reduce cerebral blood flow, and shift the oxyhemoglobin dissociation curve leftward, impairing peripheral oxygen delivery. Instead, we maintained mild, controlled hyperventilation (PaCO_2_ 30-35 mmHg) alongside pharmacologic vasodilation to balance pulmonary vasodilation with systemic organ perfusion.

Our use of a TIVA technique with high-dose fentanyl and midazolam also contributed meaningfully to the outcome. At two months of age, the myocardium is particularly sensitive to the direct depressant effects of volatile agents such as sevoflurane. High-opioid TIVA blunts the intense neuroendocrine stress response of major pediatric cardiac surgery, helping prevent abrupt catecholamine surges that could destabilize the PVR/SVR balance and precipitate a pulmonary hypertensive crisis [27].

Finally, although early (“fast-track”) extubation is increasingly favored [1], we chose a more cautious plan and continued mechanical ventilation for 48 hours postoperatively. Given the older age at presentation and the higher likelihood of reactive pulmonary hypertensive crises, maintaining autonomic stability and limiting pulmonary vasoconstriction through continuous opioid-based sedation and analgesia was considered the safest route to a stable recovery [28]. That this infant tolerated 117 minutes of cross-clamp time and 185 minutes of CPB without mechanical circulatory support (e.g., ECMO) provides strong clinical support for the idea that a large, non-restrictive PDA can preserve LV structure and function in late-presenting d-TGA.

Study limitations 

Postoperative evaluation of coronary patency and branch pulmonary artery flow was based only on transthoracic echocardiography (TTE). While TTE showed reassuring indirect signs of myocardial perfusion, we did not obtain CT angiography or cardiovascular magnetic resonance (CMR) before discharge, largely because of institutional limitations in routinely applying these modalities to fragile infants. Long-term outpatient follow-up using these higher-resolution imaging techniques remains important to exclude late, subclinical anastomotic stenosis. Additionally, as a single-case report (N=1), this study inherently demonstrates clinical feasibility and association rather than establishing a definitive causal mechanism or generalizable selection criterion. While a large PDA provided favorable loading conditions in this infant, larger prospective cohorts and multi-center studies are needed to evaluate the generalizability and long-term outcomes of delayed primary ASO in late-presenting d-TGA.

## Conclusions

A primary ASO at two months of age can be feasible when a non-restrictive PDA has preserved LV function. Looking at the bigger picture, getting a good outcome here took a lot more than just surgical skill. It required exact echo planning up front, meticulous handling of the coronaries, and an anesthesia team completely focused on controlling PVR. Obviously, a single case has its limits. We still need larger comparative cohorts to see if this delayed ASO strategy is safe for more patients and to track how they do in the long run.
